# Perspective on patient and non-academic partner engagement for the responsible integration of large language models in health chatbots

**DOI:** 10.1186/s40900-025-00804-1

**Published:** 2025-12-22

**Authors:** Nikhil Jaiswal, Yuanchao Ma, Bertrand Lebouché, Dan Poenaru, Marie-Pascale Pomey, Sofiane Achiche, David Lessard, Kim Engler, Zully Montiel, Hector Acevedo, Rodrigo Rosa Gameiro, Leo Anthony Celi, Esli Osmanlliu

**Affiliations:** 1 Department of Family Medicine, Faculty of Medicine and Health Sciences, McGill University, Montreal, QC Canada; 2 Centre for Outcomes Research & Evaluation, Research Institute of the McGill University Health Centre, Montreal, QC Canada; 3 School of Medicine, Faculty of Medicine and Health Sciences, McGill University, Montreal, QC Canada; 4 Chronic Viral Illness Service, Division of Infectious Diseases, Department of Medicine, McGill University Health Centre, Montreal, QC Canada; 5 Department of Biomedical Engineering, Polytechnique Montreal, Montreal, QC Canada; 6 Harvey E. Beardmore Division of Pediatric Surgery, Montreal Children’s Hospital, McGill University Health Centre, Montreal, QC Canada; 7 Mila - Quebec AI Institute, Montreal, QC Canada; 8 University of Montreal Hospital Research Center, Montreal, QC Canada; 9 Centre of Excellence on Partnership with Patients and the Public, Montreal, QC Canada; 10 Department of Health Policy, Management and Evaluation, School of Public Health, University of Montreal, Montreal, QC Canada; 11 Department of Family Medicine and Emergency Medicine, University of Montréal, Montreal, QC Canada; 12 MIT Critical Data, Massachusetts Institute of Technology, Cambridge, MA USA; 13 Laboratory for Computational Physiology, Harvard–MIT Division of Health Sciences & Technology, Cambridge, MA USA; 14 Beth Israel Deaconess Medical Center, Boston, MA USA; 15 Patient-Partner, Montreal, QC Canada; 16 Patient-Partner, Cambridge, MA USA

**Keywords:** Artificial intelligence (AI), Large language models (LLMs), Chatbot, Patient engagement, Co-construction

## Abstract

Uses of large language models (LLMs) in health chatbots are expanding into high-stakes clinical contexts, heightening the need for tools that are evidence-based, accountable, accurate, and patient-centred. This conceptual, practice-informed Perspective reflects on engaging patients and non-academic partners for the responsible integration of LLMs, grounded in the co-construction of MARVIN (for people living with HIV) and in an emerging collaboration with MIT Critical Data. Organised by the Software Development Life Cycle, we describe: conception/needs assessment with patient partners to identify use cases, acceptable trade-offs, and privacy expectations; development that prioritises grounding via vetted sources, structured human feedback, and data-validation committees including patient partners; testing and evaluation using patient-reported outcome measures (PROMs) and patient-reported experience measures (PREMs) chosen in collaboration with patients to capture usability, acceptability, trust, and perceived safety, alongside task performance and harmful-output monitoring; and implementation via diverse governance boards, knowledge-mobilisation materials to set expectations, and risk-management pathways for potentially unsafe outputs. Based on our experience with MARVIN, we recommend early and continuous engagement of patients and non-academic partners, fair compensation, shared decision-making power, transparent decision logging, and inclusive, adaptable governance that can evolve with changing models and standards. These lessons highlight how patient partnership can directly shape chatbot design and oversight, helping teams align LLM-enabled tools with patient-centred goals while building accountable, safe, and equitable systems.

## Abstract (plain language summary)

Health chatbots powered by large language models (LLMs) can make medical information more accessible, but most are developed without meaningful input from the people who will use them. This risks unsafe answers, hidden bias, and tools that mainly work for privileged groups. Our team built a chatbot called MARVIN to support people living with HIV, and we are now adapting it for cancer care and children’s health. Patients, caregivers, and community partners shaped what MARVIN should do, chose which sources it should trust, and tested early versions. Their feedback led to concrete improvements including clearer language, more relevant features, and safeguards against misinformation. We are also partnering with MIT Critical Data, which brings patients, members of the public, clinicians, engineers, and policymakers together at events to find and fix bias in medical AI. We have learned that technical fixes alone are not enough: trust, fairness, and accountability require active involvement of diverse users at every stage. Based on these lessons, we recommend: (1) including patients and non-academic partners from the start so their insights can shape core design decisions; (2) compensating them fairly so participation is sustainable; (3) giving them real decision-making power so their input is not tokenistic; and (4) being transparent about the limits of AI so expectations are realistic. In our experience, responsible health AI depends on the lived expertise of the people it serves.

## Introduction

Artificial intelligence (AI)-powered chatbots, capable of facilitating human-computer interactions, can add value to millions of healthcare encounters worldwide. As scalable interventions, many chatbots have shown promise for patient education, risk assessment, data collection, and self-management support [1, 2]. Building on this potential, large language models (LLMs) extend chatbot capabilities even further. LLMs hold great promise for a wide range of tasks in healthcare including answering patient questions, summarizing clinical notes, and performing numerous administrative tasks [3, 4].

Applications of chatbots based on these LLMs are increasing, highlighting opportunities and concerns over their use in high-stakes clinical settings that require evidence-based, accountable, and accurate tools, but also the imperative that such systems remain patient‑centred and designed to respect individual preferences, cultural contexts, and shared‑decision‑making goals [5–7]. Therefore, responsible integration of LLMs with a commitment to ethical economic, social, and environmental principles is crucial to optimize the value added by health chatbots [8].

Discussions around digital health, including those about AI-powered health chatbots often overlook deeper social and ethical considerations, particularly regarding the dynamics between researchers, technologists, and patients [9, 10]. In many cases, there is a distinct power imbalance when patients are from marginalized backgrounds or have fewer resources [11]. Moreover, the patients who may offer the most insightful perspectives (i.e., those with the most severe health challenges or the caregivers who support them) have limited time to participate in design processes. Consequently, essential patient voices risk being underrepresented, perpetuating gaps in knowledge that negatively impact innovation.

This Perspective is organised as follows: (1) the key challenges we face developing an AI-powered health chatbot (MARVIN), including the integration of LLMs once their availability increased; (2) what we did and learned in co-constructing MARVIN; (3) what we will do next to improve on (2), including datathons, policy camps, and an upcoming prompt-a-thon; and (4) general, practice-informed recommendations.

## Key challenges

In brief, the main challenges of LLM integration in health chatbots are: safety and accuracy (hallucinations, provenance, and model updates), equity and bias (uneven performance across groups), accountability and governance (clear decision, rights and traceability), evaluation gaps (limited use of PROMs/PREMs and transparent reporting), and trust/adoption (privacy expectations and realistic communication about limits). These motivate engagement through co-construction across the Software Development Life Cycle (SDLC).

### Patient and non-academic partner engagement

Patient and non-academic partner engagement refers to the meaningful involvement of individuals who are end-users or stakeholders in healthcare, but are not part of traditional academic research teams. This includes patients, caregivers, healthcare providers, industry partners, and members of community organizations [12]. Engaging these partners involves incorporating their insights, experiences, and preferences throughout the development process to ensure that resulting tools are relevant, inclusive, and effective.

The Montreal Model [13] is a framework for engagement that proposes a continuum of strategies for engaging patients and other members of the public. The highest level of partnership refers to co-constructing services, programs, research projects, and quality improvement initiatives with patients and other members of the public. By leveraging their lived experiences, this approach ensures that health programs and policies reflect the needs and perspectives of those they affect. Furthermore, patients and non-academic partners are involved at all stages of the development process, from formulating research questions to the interpretation and dissemination of research results. This ensures that research is patient-centred and aligned with community needs. Our recommendations are based on this framework.

We applied the Montreal Model’s co-construction principle by involving patient and non-academic partners from needs assessment through development and usability testing and by validating knowledge-base content prior to release. Progression between build stages required meeting predefined thresholds on validated usability/acceptability measures.

The capacity of patients and non-academic partners to engage in such processes varies widely [11]. Health status, socioeconomic circumstances, lack of trust in health agencies, and caregiving responsibilities can all limit participation. Community-Based Participatory Research (CBPR) aims to address these barriers and promote longitudinal engagement, as illustrated by approaches that bring together researchers and community members to co-develop and co-lead projects [14]. Power imbalances remain a significant barrier to genuine engagement. Tokenistic approaches, where patients are invited but their input is minimally integrated, undermine the potential for collaboration and can overshadow well-intentioned efforts, leading to superficial contributions rather than authentic partnership [15]. Addressing these challenges involves implementing deliberate strategies to accommodate varying schedules, provide adequate compensation, and create supportive environments where individuals are empowered to shape project design. Moreover, an ethics framework that explicitly recognizes potential power disparities can help ensure equitable involvement [16]. We next illustrate how these principles played out in practice by tracing MARVIN through the SDLC.

### Insights throughout the co-construction of MARVIN

In 2020, our multidisciplinary team applied principles of responsible innovation by successfully co-constructing and implementing an intelligent chatbot, MARVIN, specifically for people living with HIV [17]. MARVIN is currently expanding into a family of chatbots tailored to various specialties such as oncology and pediatrics, suggesting the potential for scalability [18]. The process of engagement and co-construction is described below for each phase of MARVIN’s development and expansion. For reference, MARVIN originally operated as a rule-based, decision-tree chatbot powered by natural language processing techniques, delivering scripted responses derived from vetted clinical guidelines. We are now in the Development phase of migrating to a hybrid architecture that integrates large language models (LLMs). To provide a consolidated view of the engagement process across the Software Development Life Cycle (SDLC), we summarize patient/non-academic partner roles, research team responsibilities, and observed barriers (Table 1). This table complements the narrative by mapping key decisions, timelines, and logistical insights across phases.

**Table 1:** MARVIN Engagement Matrix: Phase status, roles, decision rights, timelines, and cadence

SDLC Phase	Status	Lead(s) (per protocol)	Patient/non-academic partner decisions	Research Team decisions	Indicative timeline	Meeting / activity cadence	Limitations / barriers (observed)	Logistical insight / Learning points
Conception/Needs-assessment [18]	Completed	A trained interviewer facilitates each focus-group session	Patients articulate use-scenarios, integration topics, and preference signals in focus-groups	Primarily listening / data-gathering role; no prescriptive decision power noted at this stage	Objective 1 ≈ 4 months	Four 2-hour focus-groups with ~ 5 participants each	Representativeness; varying privacy literacy; language access; time constraints; compensation expectations.	Pre-session primers; bilingual materials; hybrid (virtual + in-person); transparent renumeration.
Development (Co-design) [18]	Completed (HIV); In-progress (Pediatrics)	Co-construction design committee (patients + HCPs + researchers) steers design	Validate content; attend three 2-hour co-construction workshops; vote on user experience changes	Engineers run iterative development workshops with the committee to test/refine prototypes	Within Objective 1’s 4-month window; real-time updates thereafter	Committee meets every 2 weeks between workshops	Large-team coordination; accessibility (captioning, device access); power asymmetries.	Smaller ad-hoc working groups; decision log + change control; accessibility checklist; co-authorship & voting to rebalance power.
Test & Evaluation (Usability-Obj. 2) [17, 18]	Achieved (HIV)	Study team enrolls 30 participants	Participants chat with MARVIN for 3 weeks, join focus-groups, complete surveys	If usability is sub-optimal, update version and rerun test	3-week pilot + week-4 focus-group window	As-needed chatting; MARVIN auto-pings if silent > 1 week	Self-selection bias; novelty effects; device/connectivity constraints; limited follow-up window.	Iterative feedback loops allowed rapid adjustments between pilot and retest; short evaluation window limited longitudinal insight.
Implementation (Open deployment-Obj. 3) [18]	Underway	Optimized chatbot released 24/7 on the web	Users choose when to chat; complete implementation-outcome questionnaire every 2 months	Monitor fidelity, adoption, usability; update model if outcomes flag issues	12-month participation period	Automated survey links every 2 months; nudges if no chat in 1 month	Reach to under-served groups; evolving privacy requirements; integration constraints.	Community partnerships for outreach.
Governance & Continuous-improvement [18]	Ongoing	Governance board reviews weekly log-audits & safety reports	Participants can flag inaccuracies; reports feed into board discussions	Developers carry out revisions of chat-logs for error interception	Continuous cycle	No fixed cadence; continuous monitoring with ad-hoc meetings	Longitudinal collaboration.	Prompt-a-thon for unique insights in how users interact with LLMs.

#### Conception/Needs Assessment

**Fig. 1:**
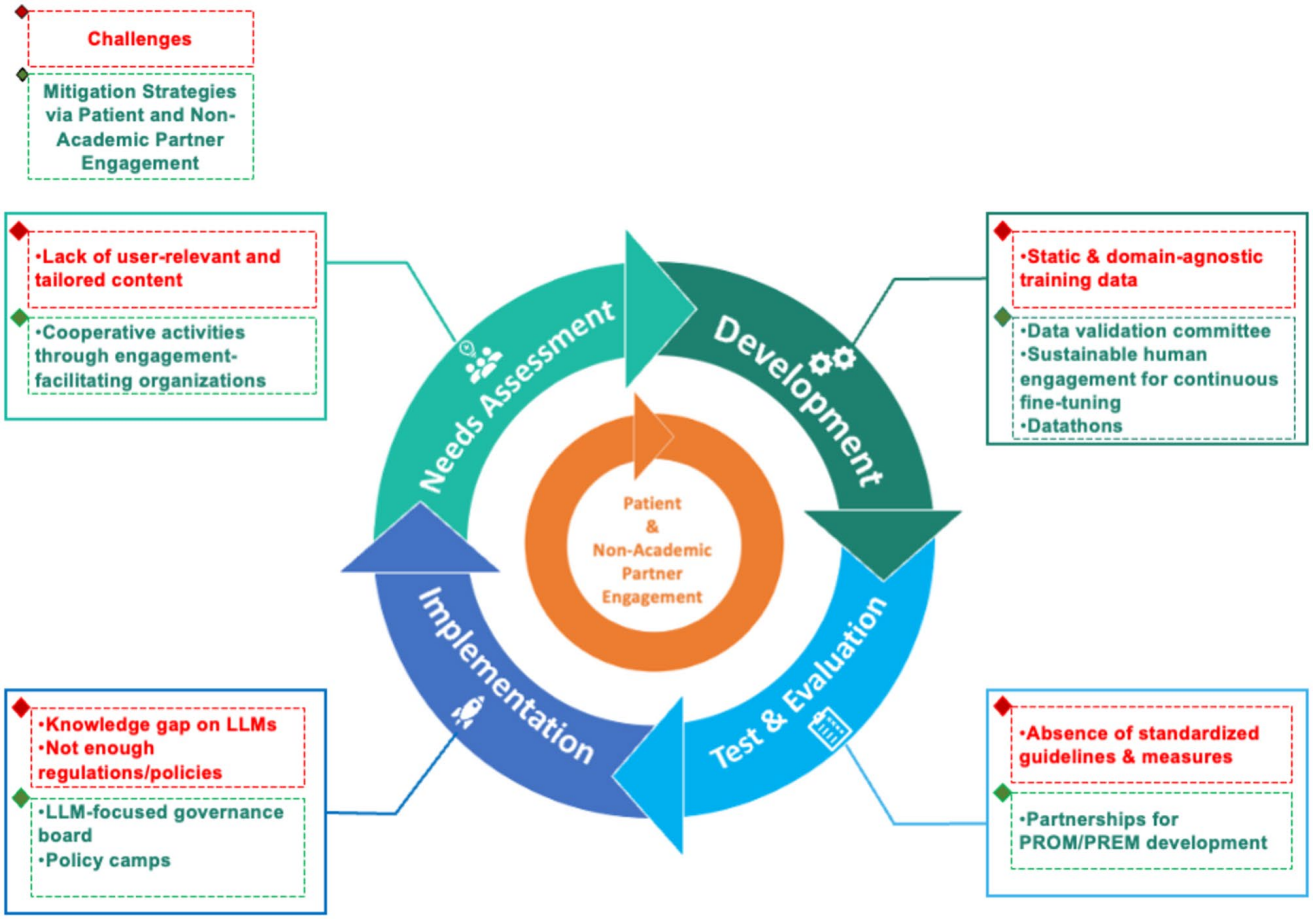
Challenges and mitigation strategies in each step of the integration of Large Language Models in the Health Chatbot Software Development Life Cycle

Engagement through co-construction benefits successful LLM integration throughout the lifecycle of healthcare chatbots (Fig. 1). In the conception stage, early engagement through brainstorming workshops and focus groups identified several features of MARVIN. Ongoing dialogues with patient experts revealed that medication adherence was a primary challenge for people living with HIV, which helped determine MARVIN’s key functionalities. Patients and healthcare providers also identified specific points in the continuum of care where a chatbot could provide the most value, such as offering reminders between clinic visits and providing information on regulations for international travel. In addition, discussions about privacy and transparency led us to consider trade-offs between open-source LLMs and more powerful proprietary ones. To operationalize these principles, for the pediatric adaptation of MARVIN (MARVIN-CHAMP), we curated PedMedQA, a pediatric question-answering benchmark, to gauge the performance of both open- and closed-source LLMs across pediatric specialties and age strata within future versions of MARVIN-CHAMP [19].

A co-construction committee, including patient-experts, pharmacists, and a physician, was established to ensure that MARVIN addressed user desires and preferences precisely. As MARVIN evolves in other clinical domains, we will continue to engage patients and non-academic partners to assess the integration of LLMs, ensuring that any new functionalities align with user needs and expectations.

#### Development

General‑purpose LLMs, trained on wide‑ranging clinical and non‑clinical corpora, often lack the domain‑specific nuance that specialised healthcare applications require; therefore, health chatbots must ground their answers in the most current, clinically validated evidence.

Following peer review principles, data validation committees comprised of patients and non-academic partners assisted in reviewing and ranking data used by MARVIN to ensure data quality and pertinence. Retrieval Augmented Generation (RAG) techniques, which retrieve the most relevant, up‑to‑date documents from a vetted medical knowledge base, reduce the hallucinatory and time-static challenges of LLM-based chatbots.

Such participation supports inclusivity (by integrating multiple perspectives in data curation), relevance (ensuring the information is medically accurate, context-specific and contains recent, evidence-based clinical practices), and effectiveness (improving chatbot performance with real-world user input).

#### Testing and Evaluation

At the testing and evaluation phase, LLM-based chatbots lack standardized and widely-adopted assessment frameworks and guidelines for transparent reporting [7]. Engagement is essential to select or develop patient-reported outcome and experience measures for health trials [20, 21].

Researchers need robust evaluation frameworks and transparent reporting guidelines that can be applied in real-world clinical settings. They also require a comprehensive understanding of user perspectives, which is especially crucial in times of fast-paced technological innovation. A prior review reported less than 10% of AI-based digital health trials include participant perspectives as endpoints-in part due to the lack of high-quality evaluation measures [22]. Partnerships with patients and non-academic partners can help contribute to the identification of relevant, user-centred outcomes and metrics to evaluate LLM-based health chatbots.

For example, during the testing phase of MARVIN, we collaborated with patient-partners to select appropriate patient-reported outcome and experience measures (PROMs and PREMs). These included assessments of usability and acceptability. Patient feedback led to iterative improvements in MARVIN's interface and functionality, ensuring that the chatbot met the needs of real-world users.

Additionally, recruitment for the testing phase surfaced equity gaps: despite outreach through clinics and community organizations, women were underrepresented in early cohorts. We recognize that convenience sampling and study logistics likely contributed (for example, reliance on clinic-based referrals and dissemination via foundations/associations) and the requirement to use a Facebook Messenger-based chatbot which may have differentially excluded some potential participants.

#### Implementation

During implementation, LLM-based healthcare chatbots face challenges related to low adoption, trustworthiness, and access. Forming a multidisciplinary and diverse governance board can help identify “blind spots” early, make design inclusive and compelling, and adapt to rapid advancements in chatbot technologies through the technical expertise of industry partners.

Knowledge mobilization through patient and non-academic partner engagement to develop educational materials about the functionalities and limitations of LLMs can help calibrate expectations. Additionally, an LLM-focused governance board can provide additional communication channels, closely track and mitigate potential ethical and technical issues, and identify risks that were not anticipated at earlier steps.

For MARVIN specifically, these educational materials and an LLM-focused governance board are being developed now, prior to implementation, to provide additional communication methods, track and mitigate potential ethical and technical issues, and identify risks not anticipated at earlier stages.

## Collaboration with MIT Critical Data

As we continue to scale MARVIN to other clinical domains, we recognize that engaging diverse communities in AI is our strongest defense against bias in healthcare. To enhance our efforts, we are collaborating with MIT Critical Data, a global initiative focused on equitable AI development through datathons and policy camps [23].

A datathon is an event that brings together participants from diverse disciplines to collaboratively analyze data and solve challenges; it is focused on patient safety and health equity by developing correction models to address health disparities affecting marginalized populations [24]. A policy camp is a program designed to analyze, discuss, and mitigate unintentional data bias in healthcare decision-making using machine learning and AI, where participants work together to understand and improve policies at all levels (e.g., institutional, local government, federal government, supra-national) governing AI algorithms in medicine.

Policy camps can help address equity and build capacity in health AI by bringing together policymakers, patient-partners, and members of the community to develop frameworks that ensure AI tools are used in a manner that is equitable.

### Prompt-a-thon

Scheduled for 2026, the inaugural Health Chatbot Prompt-a-thon, hosted within a scientific symposium of the Québec Digital Health Network, and organized in collaboration with MIT Critical Data, will elevate patient and non-academic partner voices in defining the conditions under which health chatbots deliver individual and societal value. Its explicit focus on transparency, accountability, knowledge mobilisation, and environmental sustainability distinguishes it from earlier MIT Critical Data events that centred primarily on academic, clinical, and student contributors.

Approximately 60–80 participants including patients, caregivers, community advocates, policymakers, clinicians, engineers, and social scientists, will collaborate in multidisciplinary teams, each guided by moderators. Every team will include at least one patient or non-academic partner and at least one technical or clinical contributor, ensuring balanced expertise and lived experience.

The teams will craft and iteratively refine bilingual prompts for pediatric use-cases while MARVIN generates responses in real time. Then, a jury will evaluate those responses, weighing numerous factors including clarity, cultural safety, actionability, technical soundness, and carbon footprint.

Outputs from the Prompt-a-thon will flow directly into the pediatric adaptation of MARVIN to MARVIN-CHAMP (Chatbot to Assist the Management of Pediatric patients). A curated bilingual prompt library generated will enrich CHAMP’s RAG pipeline, while the co-created evaluation rubric will be encoded as automated tests.

Methodologically, the Prompt-a-thon will employ a mixed-methods participatory-action-research design [25]. Each prompt–response cycle yields quantitative data including token counts, latency, hallucination flags, readability scores as well as qualitative data captured through audio-recorded/transcribed team discussions and jury deliberations. Descriptive statistics will map error patterns and score distributions, while reflexive thematic analysis will probe issues such as cultural safety and preferred answer formats. In this way, lessons learned during the event translate into safer, more equitable iterations of the chatbot for future pediatric care.

## Recommendations based on reflection

Based on our experience with MARVIN and insights from our emerging collaboration with MIT Critical Data, we offer practice-informed reflections and provisional recommendations to help optimize the value of engagement in chatbot co-construction. These reflections are based on experience, not on formal comparative evaluation. To make these recommendations more usable for teams with varying resources, we distinguish between essential practices that are foundational (e.g., fair compensation, early engagement, transparent decision logging) and advanced practices that may be more feasible for later-stage projects (e.g., formal data validation committees).

### Engage early and continuously (from conception through implementation)

Early and continuous engagement of patients and non-academic partners from the conception stage through implementation is essential, as their insights help identify relevant needs, determine where LLMs can be most valuable, and clarify acceptable trade-offs in model selection.

### Establish data-validation committees and transparent, adaptable governance

Establishing data validation committees that include patients and non-academic partners is also crucial for reviewing and ranking data sources, thereby ensuring data quality and pertinence. A governance structure emphasizing transparency, accountability, and adaptability bolsters this delicate mission. Moreover, the ethical participation of patients and non-academic partners in RAG pipeline development processes can help align LLM outputs with human objectives and reduce harmful outputs. These processes are currently underway for MARVIN.

### Prepare and support participants; budget for fair compensation and dedicated coordination

Great care must be exercised to adequately prepare and support participants, who may be exposed to distressing material. When deemed appropriate, participant compensation must follow guidance from organizations with expertise in patient and public engagement [26, 27]. To support meaningful and sustained collaboration with patients and non-academic partners, especially when sensitive or distressing topics may arise, projects must allocate dedicated financial resources and assign clear managerial responsibility (e.g., an engagement coordinator or patient-partnership lead) to oversee communication, ethics, and continuity of participation.

### Co-develop evaluation measures; adopt inclusive governance; understand attitudes; communicate limits transparently

We further recommend developing standardized evaluation measures, including PROMs and PREMs, in collaboration with patient and non-academic partners to ensure the relevance of health chatbot trials. Adopting inclusive governance through multidisciplinary and diverse boards can address ethical considerations, uncover blind spots, and foster inclusive design. In addition, it is important to better understand how patients and non-academic partners feel about the increasing use of AI in their care, as this may reveal underlying barriers to participation and guide more responsive engagement strategies. Finally, transparent communication about how AI technologies function—including limitations such as hallucinations and biases—builds trust and empowers users to engage critically with chatbot technology.

### Confront power imbalances and avoid tokenism

Above all, we must confront uncomfortable truths about current forms of patient and non-academic partner engagement. First, the balance of power often tips in favor of researchers and technologists, unless patients have considerable privilege themselves. Second, even when invited to participate, patients frequently lack the sustained agency and resources required for meaningful engagement. Third, and perhaps most paradoxically, those most in need (e.g., individuals who are very ill or caregiving under intense pressure) are often least able to devote time to the design process. Ignoring these realities risks turning engagement into a superficial exercise that benefits neither science nor health equity. There is a subtle yet crucial distinction between involving patients because their insights are scientifically indispensable and doing so merely out of obligation. By embracing the former approach and actively challenging the mindset that frames patients as lacking, we can move closer to a truly responsible, patient-partnered model of innovation.

## Limitations

This narrative is practice-informed and descriptive rather than comparative or experimental, and its claims should therefore be interpreted as reflections and provisional recommendations rather than empirically demonstrated effects. Although MARVIN is being adapted to oncology and pediatrics, the engagement strategies, governance mechanisms, and technical decisions we describe have not yet been prospectively evaluated in these domains. Moreover, the LLM technologies and evaluation standards are evolving rapidly. Our collaboration with MIT Critical Data is emerging; we have not yet shown how datathons, policy camps, or prompt-a-thons measurably alter design decisions, governance, or clinical outcomes.

## Conclusion

LLM‑enabled health chatbots will advance patient‑centred care if their design, evidence base, and oversight are shared with patients and non‑academic partners from the outset. Using MARVIN as our case, we showed how co‑construction can shape needs assessment, development, testing, and implementation. Our emerging collaboration with MIT Critical Data, including an upcoming Prompt-a-thon, extends this model by aiming to democratize technical literacy, surface bias, and inform governance.

From these reflections we offer recommendations: engage early and continuously; compensate fairly; minimize participant distress; share real decision‑making power; document choices transparently; and institutionalize inclusive, adaptable governance that can evolve with rapidly changing models and standards.

## Data Availability

No datasets were generated or analysed during the current study.
